# Label-free viability assay using in-line holographic video microscopy

**DOI:** 10.1038/s41598-022-17098-y

**Published:** 2022-07-26

**Authors:** Rostislav Boltyanskiy, Mary Ann Odete, Fook Chiong Cheong, Laura A. Philips

**Affiliations:** Spheryx, Inc., New York, NY 10016 USA

**Keywords:** Optical imaging, Microscopy, Cell death

## Abstract

Total holographic characterization (THC) is presented here as an efficient, automated, label-free method of accurately identifying cell viability. THC is a single-particle characterization technology that determines the size and index of refraction of individual particles using the Lorenz–Mie theory of light scattering. Although assessment of cell viability is a challenge in many applications, including biologics manufacturing, traditional approaches often include unreliable labeling with dyes and/or time consuming methods of manually counting cells. In this work we measured the viability of *Saccharomyces cerevisiae* yeast in the presence of various concentrations of isopropanol as a function of time. All THC measurements were performed in the native environment of the sample with no dilution or addition of labels. Holographic measurements were made with an in-line holographic microscope using a 40$\times$ objective lens with plane wave illumination. We compared our results with THC to manual counting of living and dead cells as distinguished with trypan blue dye. Our findings demonstrate that THC can effectively distinguish living and dead yeast cells by the index of refraction of individual cells.

## Introduction

The use of yeast cells, particularly *Saccharomyces cerevisiae*, is ubiquitous in both industry and academia^1–3^ for applications from cell-based experiments to protein manufacturing. For example, in medical research yeast serves as a model organism to study genetic mutations relevant in cancer^4–6^. In biopharmaceutical research and manufacturing, yeast cells are employed as mini-factories for the production of proteins of interest^7–9^. Additionally, in one of the most well-known applications, yeast is used in consumer product research and manufacturing to optimize beer, wine and bread-making^10–13^.

Cell viability is a key parameter of interest in many of these applications^14^. While numerous methods of measuring viability of yeast exist, most share one limitation: the need to stain cells with a dye. Dye-based methods, such as the trypan blue (TB) exclusion assay, rely on healthy cell membranes being impermeable to the dye while the membranes of dead or damaged cells allow the dye to diffuse into the cell^15^. Although this method is useful, it usually involves tedious sample preparation and manual cell counting. Manual cell counting suffers from low statistics and is prone to human error. Furthermore, the TB exclusion assay has been shown to overestimate viability and be unreliable for certain cell samples with a viability less than 70%-80%^16–18^.

Additionally, with any dye-based staining viability measurements, there is a danger that the dye can interact either with the cells or with another experimental variable in unintended ways. As an example, trypan blue has been shown to adversely interact with cells, often rupturing them and thus rendering viability measurements unreliable^19^. Although the shortcomings of label-based viability assays are well known, few label-free alternatives exist^20^.

In this work we introduce a label free, automated technique to reliably measure the viability of yeast using Total Holographic Characterization^®^ (THC). THC is a technology, based on holographic video microscopy, developed to detect, count, and characterize subvisible particles in suspension^21^. Here we assessed yeast viability using xSight, which implements THC and combines holographic microscopy with microfluidic sample handling to provide precise measurements of particle size and refractive index. The use of single-use microfluidic sample chips ensures that there is no cross-contamination between samples and no cleaning is required. Each measurement is automated and takes approximately 15 min to complete.

Holographic imaging has been utilized with living cells in previous studies^22–35^. Several studies measured and tracked important cell biophysical parameters, such as cell volume, cell thickness and dry mass^22,23,28,31,32^. The limitation of these approaches, however, is the need for media exchanges and manipulation of media osmolarity to stimulate changes in cell volume and refractive index. The present work performs measurements in the native environment of the cells without any need for media exchanges or modifications. Significant advancement has also been made in measuring the refractive index of individual mammalian cells using quantitative phase imaging with digital holographic microscopy^31,32^. In these studies, refractive index was correlated with cell size and used to distinguish two human pancreatic cancer cell lines. In the studies that measured whole cell refractive index, however, no correlation to cell viability was reported. Those who have assessed cell health using holographic techniques have done so in mammalian cells under specific conditions such as *P. falciparum* infections^29^, exposure to cadmium^30^, exposure to a variety of organic nanoparticles^33^ and other toxic compounds^34,35^. In this work we measure whole cell refractive index of yeast in a single medium in its native environment. We show that refractive index of cells can be rapidly determined with THC and used to accurately assess viability with this novel, label-free, automated technique.

To demonstrate THC as a new technology for viability studies, we used alcohol to gradually kill yeast cells, as has been done in previous yeast viability studies^36,37^. We show the viability changes of *Saccharomyces cerevisiae* under various concentrations of isopropanol and compare our results with staining using trypan blue.

## Results

### Holographic approach to yeast viability

**Figure 1:**
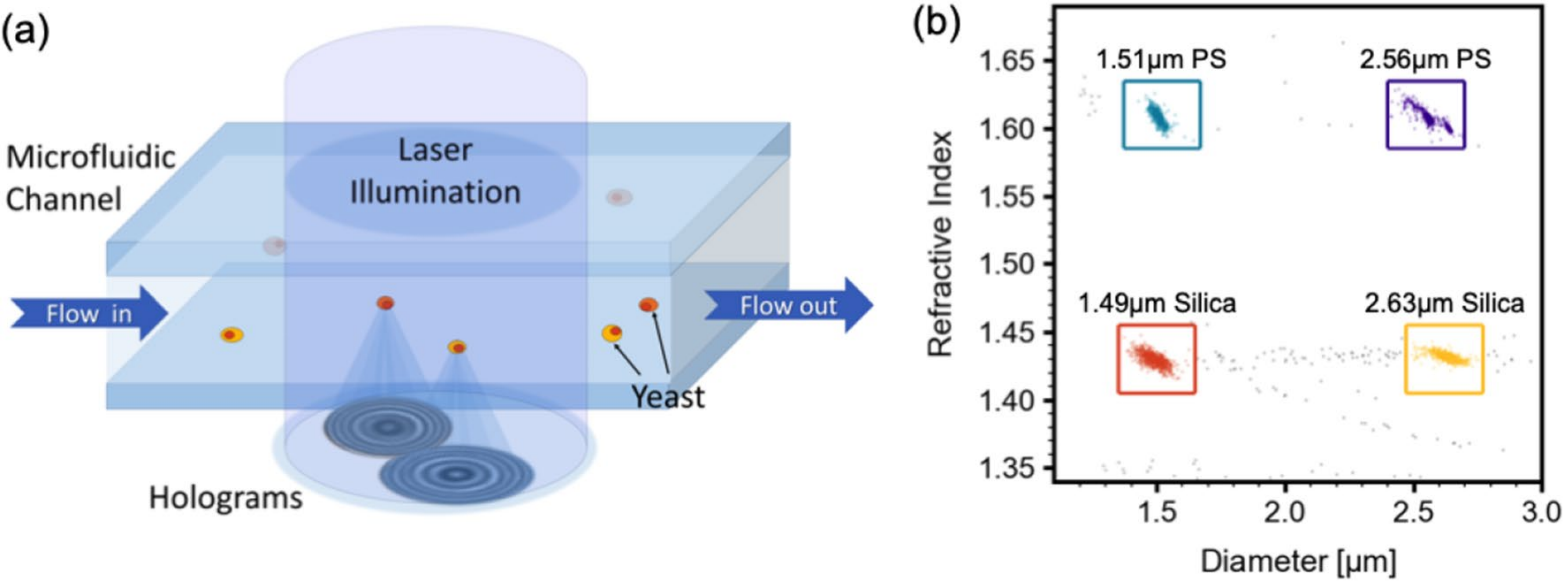
(**a**) A schematic of holographic video microscopy: as cells flow through a microfluidic chip, they are illuminated by a laser beam. Light scattered by the particles interferes with the incident light, forming holograms which are recorded on a camera. (**b**) A scatter plot of size on the horizontal axis and refractive index on vertical axis for 4 species of particles: $1.51\,\upmu$m diameter polystyrene spheres (in cyan), $2.56\,\upmu$m diameter polystyrene spheres (in violet), $1.49\,\upmu$m diameter silica spheres (in orange), $2.63\,\upmu$m diameter silica spheres (in yellow). Each point on the plot represents a single particle detected with THC during measurement. The colored boxed are user-defined regions of interest. Particles outside of the 4 user-defined boxes are colored gray.

We employ holographic video microscopy^21,38–47^ to assess yeast viability. In our approach, particles in suspension (such as yeast cells) flow through a microfluidic chip as they are illuminated by a collimated laser beam. Laser light scattered by the particles interferes with the incident laser light, forming an interference pattern called a hologram. A schematic of this technology is shown in Fig. 1a.

The holograms are recorded on a camera and fit to Lorenz–Mie theory of light scattering^40,48–50^. A fast, multi-parameter optimization of the fit then yields various particle parameters including particle size, refractive index, and the 3-dimensional position that correspond to that hologram. We do not reconstruct the 3-dimensional image of each particle, but extract morphological information about each particle from the symmetry of its 2-dimensional hologram. A key advantage of this approach, based on holographic video microscopy, is that in addition to particle size, THC quantifies the refractive index of each particle, which is indicative of its composition. For example, Fig. 1b shows a scatter plot of a sample that consists of a mix of four different particles species: two sizes of polystyrene microspheres and two sizes of silica microspheres. Each point on the plot represents a single particle detected by xSight during measurement. On the horizontal axis is the measured particle diameter and on the vertical axis is the measured particle refractive index. The colored boxes are user-defined regions of interest corresponding to different species of particles. The points are colored according to which region they fall into. From the scatter plot, one can easily observe 4 main particle distributions with two at a refractive index of approximately 1.61 in cyan and violet and two at a refractive index of approximately 1.43 in orange and yellow. Those pairs of distributions are polystyrene and silica microspheres, respectively. The refractive index of polystyrene in water when illuminated with a blue laser is 1.61 and a refractive index of 1.43 is consistent with porous silica. It is important to note that with a standard particle sizer, only two populations would be visible in this sample: one at a particle diameter of around $1.5\,\upmu$m and one at a particle diameter of around $2.6\,\upmu$m. Since THC measures refractive index in addition to size, particles of the same size but different composition can be easily distinguished, such as the $1.49\,\upmu$m silica and $1.51\,\upmu$m polystyrene spheres (in orange and cyan respectively) and the $2.63\,\upmu$m silica and $2.56\,\upmu$m polystyrene spheres (in yellow and violet respectively).

This technology has similarly been used to distinguish bacteria from plastic beads and oil in water^51^ since each of those species is composed of materials of different refractive indexes. A study of porous silica particles showed that THC is able to detect subtle changes in particle composition^52,53^. The ability to detect small changes in particle composition is due to the effective sphere model and the effective medium theory^52,54^. THC characterizes each particle that flows through the viewing region of the microfluidic chip and analyzes it assuming that it is a sphere. THC finds an effective sphere with a hologram most similar to the hologram of the particle being analyzed. When an individual particle’s composition is heterogeneous, such that various components of a particle have different indexes of refraction, the THC fitting algorithm will produce a refractive index for that particle that is an average of the refractive indexes of its various components. In the example of a porous silica particle in water, the refractive index of a given particle will be somewhere between the refractive index of the silica matrix and the refractive index of the water that fills its pores. To what extent the resulting effective refractive index will be closer to silica or closer to water depends on the particle’s porosity. Hence it is possible to extract morphological information from the behaviour of the particle refractive index in media of varying refractive indexes. Quantitative morphological information about particle porosity has been studied in porous plastics, protein aggregates, and nanoparticle agglomerates^52,55^. Additionally various particle shapes have been imaged and modeled with THC including colloidal fractals^56^, porous spheres^52,53^, rod-shaped *E. coli*^51^, microsphere dimers^57^, and transparent disks^58^. In our approach, while the scattering of each particle is fit to a model of a sphere, non-spherical particles of diverse shapes are suitable for analysis with THC.

Yeast cells are also heterogeneous particles with various cell components acting as scattering materials with different refractive indexes. Since cell death often involves physiological changes in cell structure and composition, these changes are expected to alter the refractive index and the shape of the cells, and therefore should be detected by analysis with THC.

### Distinguishing live and dead cells by refractive index

**Figure 2:**
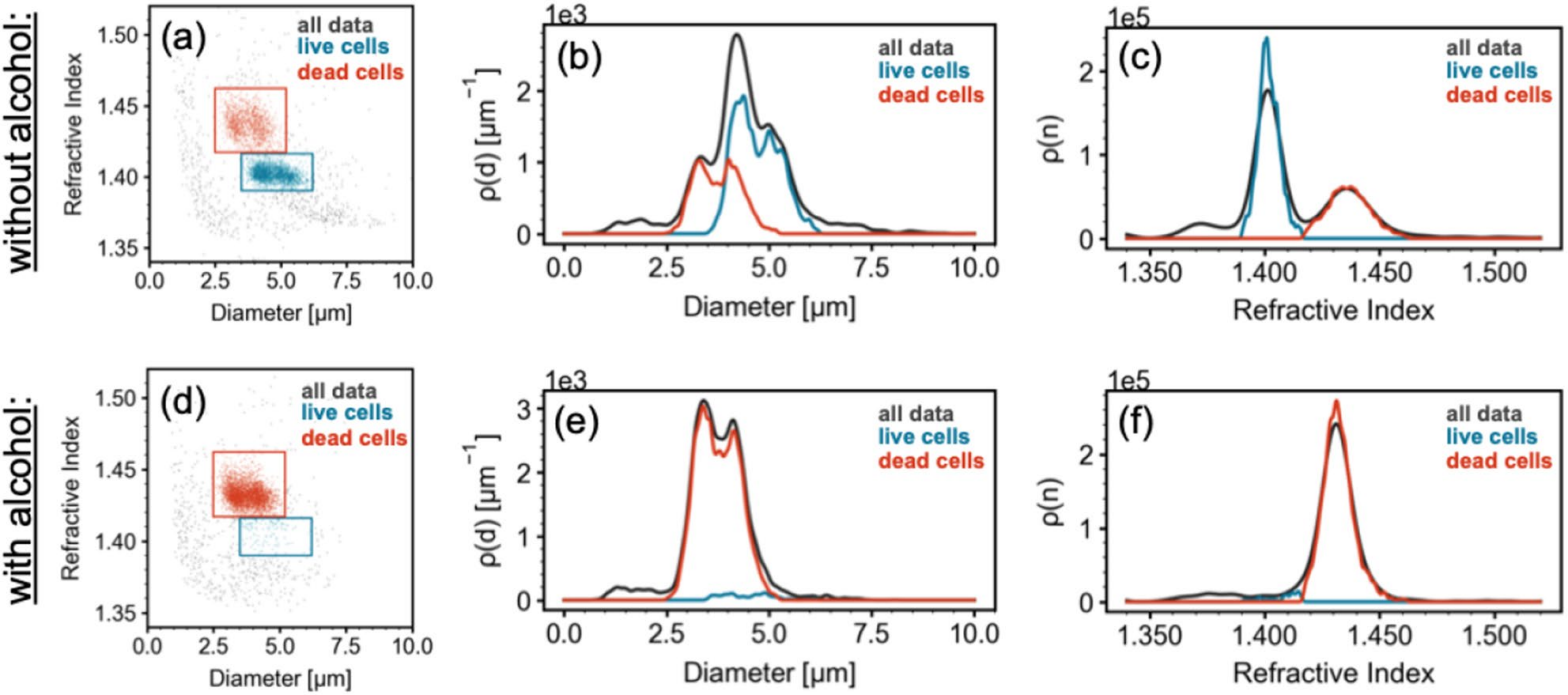
(**a**) A scatter plot of size on the horizontal axis and refractive index on vertical axis for a yeast sample before the addition of alcohol. Each point on the plot represents a single particle that flowed through the viewing region of the microfluidic chip during THC analysis. The colored boxed are user-defined regions of interest. The points colored in orange represent dead yeast cells and the points colored in cyan represent live yeast cells. Particles outside of the user-defined boxes are colored gray. (**b**) Density distributions of particle size for the sample shown in (**a**). The orange, cyan and gray curves represents the size density distributions of dead cells, live cells, and all particles respectively. The area under each curve for a given size range represents the number of particles (of the species represented by that curve) in that size range. The peak of each curve shows the most common size of each particle type. (**c**) Density distributions of particle refractive index for the sample shown in (**a**). The coloring is the same as in (**b**). The area under each curve for a given refractive index range represents the number of particles (of the species represented by that curve) in that refractive index range. The peak of each curve shows the most common refractive index of each particle type. (**d**)–(**f**) Scatter plot, size density plot, and refractive index density plot as in (**a**)–(**c**) but for a yeast sample that was exposed to 15% isopropanol by volume for 71 min.

A typical scatter plot of THC results from a yeast sample before introduction of alcohol is shown in Fig. 2a. The orange and cyan boxes are user-defined regions corresponding to dead and live yeast cells respectively. The points are colored according to which region they fall into. The orange points correspond to particles identified as dead cells, the cyan points correspond to particles identified as live cells and the gray are other particles identified as debris (see Discussion below). In the sample, shown in Fig. 2a, 51.4% of all detected particles are identified as live cells, 29.7% are identified as dead cells and 18.9% as debris.

As can be seen in Fig. 2a, while the live and dead cells overlap in size, their refractive indexes are distinct. In this sample live cells were found in the size range of $3.5\,\upmu$m to $6.2\,\upmu$m and refractive indexes between 1.39 and 1.416. Dead cells appeared at slightly smaller sizes: 2.5–5.2$\upmu$m and significantly larger refractive indexes: between 1.417 and 1.462. The overlap in size and distinction in refractive index of live and dead cells is further illustrated in Fig. 2b,c. These plots are size and refractive index density distributions respectively. The size density curve in Fig. 2b, for example, is related to the probability for a particle to have a particular size. The area under the curve over any particle size range represents the number of particles that can be found in that size range. These curves are more informative and more reliable alternatives to histograms. In these graphs, the gray curves represent all particles, the orange curves represent dead cells, and the cyan curves represent live cells. In Fig. 2b the overlap of the orange and cyan curves indicates that many live and dead cells have the same size. The overlap of the size distributions shows that identifying viability by cell size alone is not reliable. Based on Fig. 2c, the orange refractive index density curve and the cyan refractive index density curve show minimal overlap suggesting that live and dead yeast cells are distinguishable by the index of refraction. In Fig. 2c, the peak of the cyan curve is near 1.4 indicating that the most common refractive index of live cells is around 1.4 while the peak of the orange curve is near 1.435 indicating that the most common refractive index of dead cells around 1.435.

Figure 2d–f show results of the same yeast sample but 71 min after the addition of isopropanol at 15% by volume. As seen in Fig. 2d, the majority of points are orange, indicating that most of the cells have died with this exposure to alcohol. In this sample, only 2.9% of all detected particles are identified as live cells, 82.4% are identified as dead cells and 14.7% as debris. This result is represented by Fig. 2e, f where the gray curves and the orange curves overlap suggesting that the majority of identified particles correspond to dead cells. While the cyan curve is present and dominant in Fig. 2c, it is almost entirely missing in Fig. 2f confirming the dearth of live cells in a 15% isopropanol solution after 71 min.

### Comparing viability results using holographic imaging and the TB exclusion assay

**Figure 3:**
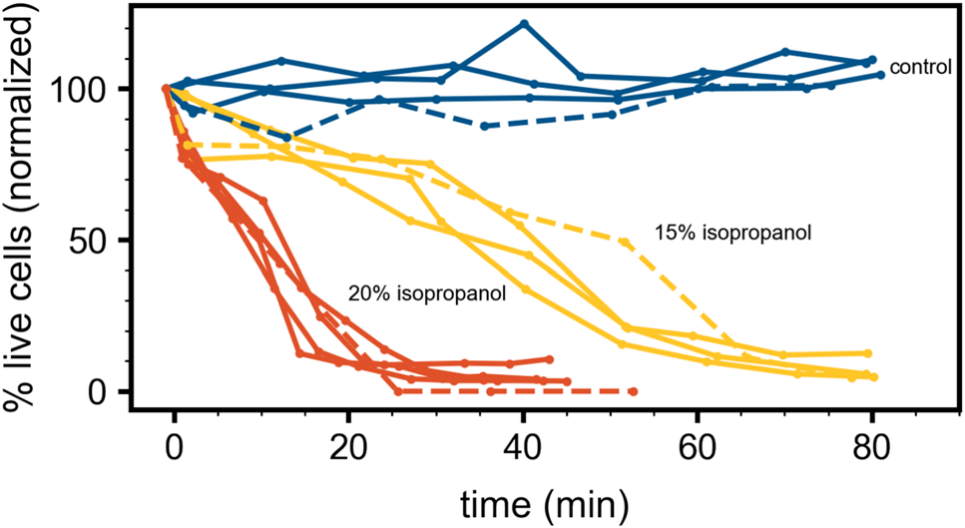
The horizontal axis represents time after the addition of alcohol. The vertical axis represented the percentage of live cells normalized by the initial time point before alcohol was added. All solid curves are measurements with THC and all dashed curves are staining measurements with trypan blue. The blue curves represent results from the control samples with no alcohol. The yellow curves represent results from samples with 15% isopropanol by volume. The orange curves represent results from the samples with 20% isopropanol by volume.

To explore how yeast respond to different concentrations of isopropanol over time and to compare our holographic approach to the TB exclusion assay, we exposed yeast samples to 0%, 15% and 20% isoproponal by volume, and measured the resulting viability over time using both xSight and trypan blue. Figure 3 shows the results of these studies.

In the graph in Fig. 3, the horizontal axis is the time after alcohol was added to the yeast mixture and the vertical axis is the percentage of live cells normalized by the live percentage before the alcohol is added. In Fig. 3 the solid curves represent measurements with THC and the dashed curves are measurements made with the TB exclusion assay. The blue curves correspond to normalized viability of yeast samples without alcohol. While some fluctuation in those curves is observed, all curves are similar to each other and show no loss of viability over the course of 80 min. The yellow curves represent results for samples with added 15% alcohol by volume. A similar decrease in normalized viability over time is seen in all of the yellow curves. The orange curves represent results for samples with added 20% alcohol by volume and show a faster drop in viability as a function of time. Across all conditions, the dashed curves are similar to the solid curves suggesting strong agreement in normalized viability changes between THC and the TB exclusion assay.

Note that while normalized viability results are consistent between THC results and TB staining results, the absolute viabilities can vary. In fact, we find a consistent overestimate of absolute viability using TB as compared to holographic results. Six consecutive measurements of a yeast sample with THC yielded an average viability of $52.3 \pm 0.9 \%$. The same sample measured with TB staining resulted in an average viability of $56 \pm 3 \%$. In addition to a higher viability measurement, consistent with an undercounting of dead cells, TB staining also showed a higher variability between measurements. This observation is consistent with other studies that reported such limitations in the TB staining approach^16^.

### Traditional staining assay is often inconclusive

**Figure 4:**
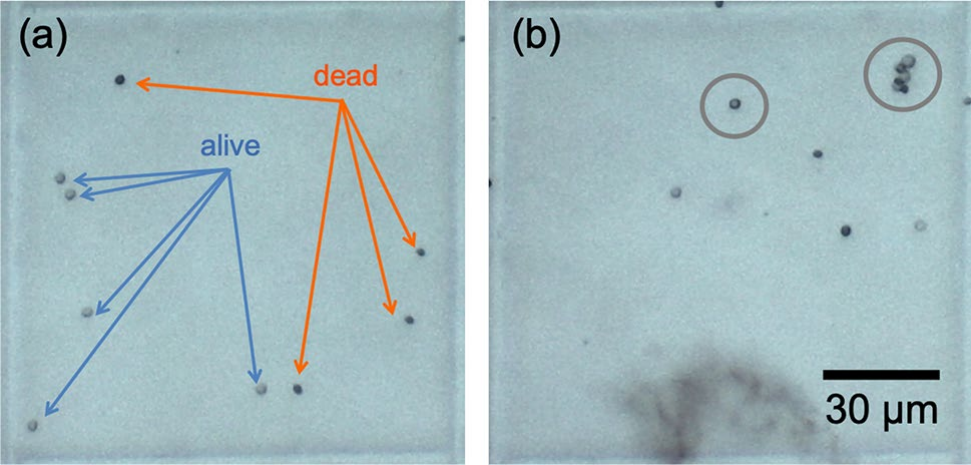
Typical results of a trypan blue exclusion assay. (**a**) A grid cell showing a mix of live and dead yeast cells. Live cells do not absorb the dye and appear light gray. They are identified with blue arrows. Dead cells are permeable to the dye and appear dark blue/gray. They are identified with orange arrows (**b**) A grid cell showing cells with an inconclusive viability status based on the trypan blue exclusion assay. Those with an inconclusive status are circumscribed by gray circles.

Staining of *Saccharomyces cerevisiae* with TB showed a mixed population of live and dead cells prior to treatment with alcohol. As shown in Fig. 4a, in a single grid, a combination of live cells (unstained and labeled in blue) as well as dead cells (stained and labeled in orange) are visible. While the viability categorization is clear in Fig. 4a, it is less clear in Fig. 4b, particularly in the cases circled in gray. Cells that partially absorb the dye and those that cluster together pose a challenge for staining-based viability assays.

The presence of ambiguously stained cells, as in Fig. 4b can also contribute to the above-mentioned higher variability in staining measurements than in measurements with THC.

### Morphological changes in cells detected with THC

**Figure 5:**
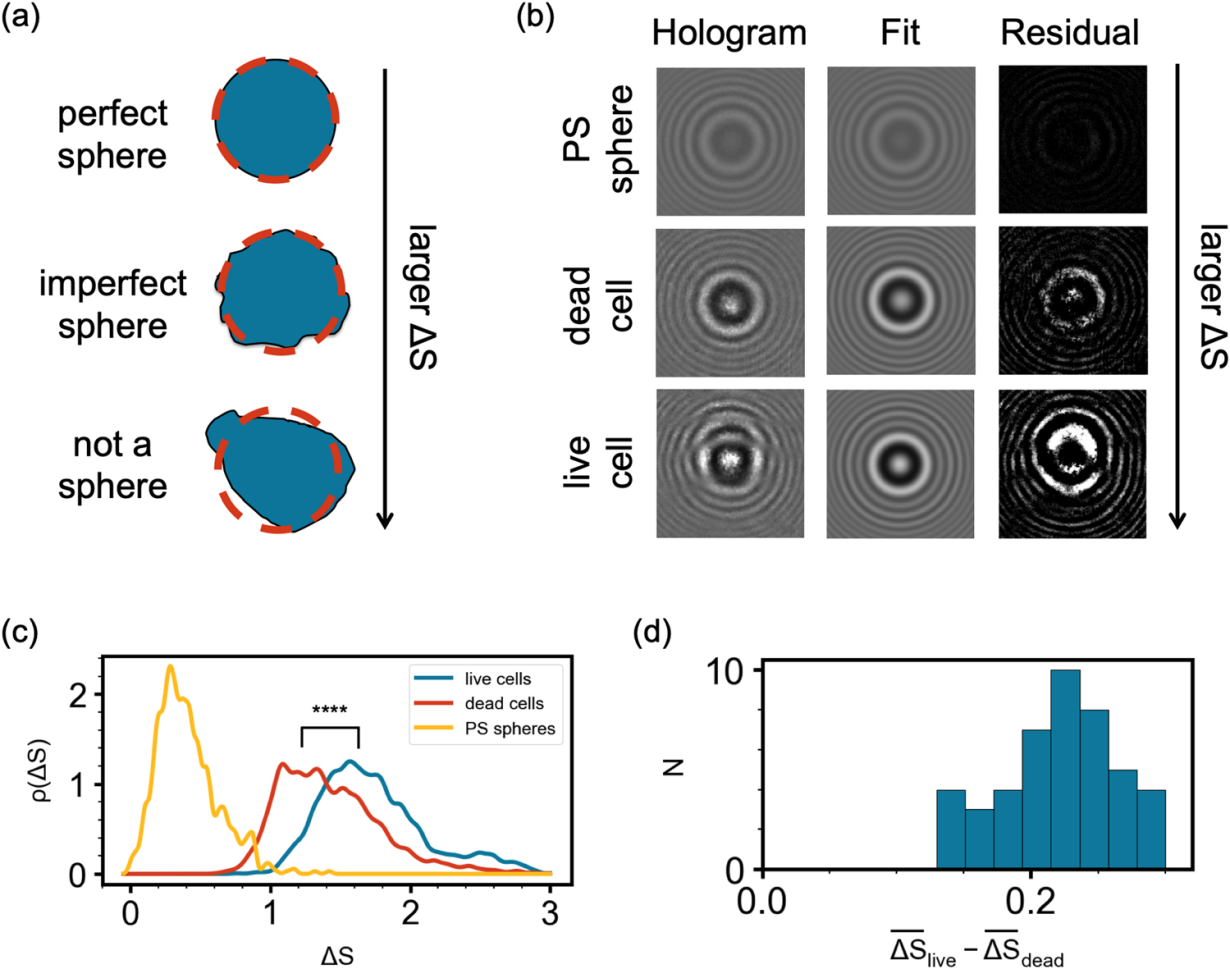
(**a**) Schematic of particles with increasing deviation from symmetry top to bottom. The orange dotted circle represents the estimated sphere that most closely approximates the given particle. (**b**) Holograms, fits to Lorenz–Mie theory, and residuals (left to right, respectively) of a polystyrene sphere, a dead yeast cell, and a live yeast cell (top to bottom, respectively). (**c**) Probability density distributions of deviation from symmetry, $\Delta S$, of $1.54\,\upmu$m polystyrene spheres (in yellow), dead yeast cells (in orange), and live yeast cells (in cyan). (**d**) A histogram of differences between the means of $\Delta S$ of live and dead yeast cells for 45 sample measurements.

In addition to measuring particle size and refractive index, THC also analyzes particle morphology. More specifically, the symmetry of the hologram corresponding to a given particle is indicative of the symmetry of the particle itself. A perfectly spherical particle is expected to have a nearly perfectly symmetric hologram, while particles that deviate from spheres show deviations from symmetry in their holograms. These deviations from the expected symmetry, $\Delta S$, are analyzed and quantified with THC. Figure 5a below shows a schematic of three particle shapes with increasing deviation from symmetry, $\Delta S$. In Fig. 5b, the first column shows examples of holograms of a 1.54 $\upmu$m polystyrene sphere, a dead yeast cell and a live yeast cell. The second column of Fig. 5b shows the fits of each of those holograms to Lorenz–Mie theory of light scattering. The third column shows the residuals between the holograms and their respective fits. The $\Delta S$ metric is in turn computed based on the residuals from the fits. Higher residuals correspond to greater deviation from symmetry and contribute to higher $\Delta S$ values.

Morphology analysis of live and dead yeast cells with THC shows that dead cells are more spherical than live cells. Figure 5c shows three probability density distributions of $\Delta S$: that of polystyrene microspheres in yellow, that of dead yeast cells in orange, and that of live yeast cells in cyan. The symmetry deviations of polystyrene microspheres are closest to $\Delta S = 0$ indicating a high degree of spherical symmetry as expected. The orange curve, corresponding to dead yeast cells, contains 2295 cells and is farther from $\Delta S = 0$, while the cyan curve, corresponding to live cells, contains 1916 cells and is farthest from $\Delta S = 0$. The $\Delta S$ means are significantly different ($\hbox {p}<0.0001$) between the live and the dead cell populations. The result that $\Delta S$ is smaller for dead cells than live cells indicates that dead yeast cells are more spherically-symmetric than live cells. Figure 5d shows a histogram of differences between the mean $\Delta S$ for live cells, $\overline{\Delta S}_{live}$, and the mean $\Delta S$ for dead cells, $\overline{\Delta S}_{dead}$. The histogram contains data from 45 samples of yeast exposed to various concentrations of alcohol. Since $\overline{\Delta S}_{live} - \overline{\Delta S}_{dead} > 0$ for each sample, dead yeast cells appeared more spherical than live cells in each measurement. While this finding in yeast has not been reported previously, such morphological changes have been identified in cancer cells exposed to pH changes that lead to their cell death^59^ and spherical shape has been correlated with cell death in *E. coli*^60^.

## Discussion

Our results indicate that yeast cell viability can be effectively assessed using THC. On average yeast cells reduced in size and increased in refractive index upon exposure to high concentration alcohol and upon eventual cell death. While we observe an average decrease in the size of dead cells compared to live ones, size alone is insufficient to distinguish the live and dead populations. Our results demonstrate that the cell index of refraction is a more effective distinguishing identifier of cell viability. This conclusion is also consistent with morphological cell changes. If cells shrink due to expulsion of low index of refraction water and the remaining cell sub-components have a higher index of refraction than water, then the net index of refraction of the cell will increase as observed in our measurements. THC characterizes each particle that flows through the xSight microfluidic chip and analyzes it as a sphere. The multi-parameter optimization finds an effective sphere with a hologram most similar to the hologram of the particle being analyzed^52,54^. A smaller cell with stronger scattering components will be identified as a smaller particle with an increased refractive index. This scenario is consistent with our results. Similar changes in optical properties of cells in response to external stress, such as osmotic pressure, have been reported in other studies^22,31^.

In addition to assessing viability, our holographic approach is also a promising way to study the progression towards cell death or other stress responses. Since many morphological changes can manifest as changes in cell size and refractive index, THC can be a powerful tool to quantitatively track those changes in real time. For example, a number of studies have explored the use of holography to determine the toxicity of various compounds by observing biophysical changes in mammalian cells^29,30,33–35^. Our approach, using THC, is an exciting complimentary tool to existing technologies. It may offer a fast assessment of changes in size and refractive index of living cells in their native environment in response to the toxins of interest.

In addition to identifying live and dead cells, our technology identified a third population of particles labeled in gray in Fig. 2a,d). Those particles were largely smaller or lower index than what we identified as yeast cells. It is possible that those are cell fragments. They could also be aggregates either of materials produced by the yeast in solution or contaminants from other sources. The combination of size and refractive index of many of these particles is consistent with what has been observed for protein aggregates with THC^55,61,62^. Further research is needed to conclusively identify those particles.

In this work we have explored the response of *Saccharomyces cerevisiae* to various concentrations of isporpopanol. Other studies have investigated cell viability in response to stresses such as other alcohols, osmotic pressure, and heat^36,37,63,64^. THC is a promising approach to study viability under those conditions as well. It offers a complimentary approach to other developing technologies that utilize other scattering techniques. Holographic changes in cells upon death should similarly be observed in other cell types such as bacteria and animal cells. THC should be a useful tool to study a wide range of biological systems.

## Conclusion

Our results present a powerful approach to assessing yeast viability using holographic video microscopy. Cell death in response to alcohol exposure was concurrent with a population increase in cell refractive index suggesting that this optical parameter is effective in identifying live and dead cells. The proposed holographic approach is fast, automated, and label-free, eliminating many of the limitations of traditional staining-based viability assays. The methodology is objective and does not require user identification of each cell once refractive index regions are identified. Therefore, THC can be extended to high-throughput, automated viability analysis in a variety industrial applications.

## Methods

### Yeast preparation

The yeast solution was made using glucose (SIGMA brand, item G8270-1KG), filtered deionized (DI) water, and dry instant yeast (Amish Market store brand, New York, NY). A glucose stock mixture was made by mixing 25g of DI water with 0.5g of glucose until the glucose was fully dissolved. Once dissolved, the glucose mixture was filtered with a 60cc luer lock disposable $0.45\,\upmu$m syringe filter (Millex brand). If not made for immediate use, the glucose mixture was stored in a $4\,^{\circ }$C refrigerator.

The glucose solution was diluted with filtered DI water in a 1:1 proportion, using 4mL of each, in a 30mL vial. Using dry instant yeast, 4–7 pellets were placed into the diluted glucose mixture and the vial was shaken by hand and vortexed until all the yeast pellets were dissolved. The yeast solutions was incubated (Benchmark, Roto-Therm mini) at $40\,^{\circ }$C. Once the incubator was at temperature, the yeast mixture was placed into the incubator for 30 min. After 30 min, the mixture was removed from the incubator and shaken briefly to dissolve any remaining yeast. A fresh yeast mixture was made for each day of experiments.

Three alcohol concentrations were tested: 0% isopropanol (control), 15% isopropanol by volume in glucose solution, and 20% isopropanol by volume in glucose solution. Each alcohol concentration was prepared by placing the solution in a 10mL vial and bringing the final volume to a total of 2mL. For the control and for 15% isopropanol solution, the samples were measured over approximately 90 min. For the 20% isopropanol solution, the samples were measured over approximately 45 min. For the control, the stopwatch was started after the solution was vortexed. For the 15% and 20% alcohol solutions, the stopwatch was started ($t=0$) once the alcohol was placed into the 10mL vial with the yeast solution. For each condition, the viability was measured once before the alcohol was introduced and then consecutively after the addition of alcohol.

### Holographic Video Microscopy

Holographic video microscopy^21,40,62^ measurements were made using xSight (Spheryx, Inc.), Spheryx’s implementation of THC. For each measurement, a $30\,\upmu$L volume of sample was placed into one of the eight reservoirs on an xCell, a disposable microfluidic sample chip (Spheryx, Inc.). Flow of the sample through the microfluidic channel was established automatically in xSight by application of a vacuum seal and a pump, creating a uniform Poiseuille flow^21,57^. A collimated laser beam with a vacuum wavelength of 638 nm illuminated the viewing region of the xCell while the sample flowed. Light scattered by particles in the sample interfered with incident light creating holograms that were recorded on a camera for further analysis. Each hologram was analyzed with THC resulting in a multi-parameter fit for the size, refractive index, and the three-dimensional position of each particle.

For each sample, sample viscosity and refractive index of the medium (water with glucose) were measured and entered into xSight. The refractive index of the medium was measured with a hand-held refractometer (Antago, pocket refractometer). Pressing the START button initiated the measurement. The measurement ensues automatically and lasts approximately 15 min for each sample.

The first measurement of the yeast solution, before the addition of alcohol, was labeled as time point “$t=-1$” and was used to normalize the rest of the viability curve. All other time points were recorded as when the START button was pressed. The volume measured for each sample was $1\,\upmu$L.

The boxes that circumscribed the live and dead yeast populations were user-defined regions of interest enabled by the xSight software. Once the regions of interest were defined, the results of the live and dead cell analyses were automatically computed producing the concentration, the median size and the median refractive index of each population. The regions were determined to delineate the distinct populations. The viability of each sample was then recorded based on the automatically computed statistics of each region.

### Staining measurements with trypan blue

Staining measurements were prepared in micro-centrifuge tubes (uLab Scientific). $30\,\upmu$L of yeast sample and $30\,\upmu$L of the dye were mixed into the tube and allowed to stand for 2 min. After 2 min, $30\,\upmu$L of the dyed sample was placed onto an Improved Neubauer Hemocytometer (Fristaden Labs). A cover slip was placed on top of the hemocytometer and viewed under a microscope (AmScope) with 10x magnification. On the hemocytometer, the cells on eight of 4x4 grid boxes were counted for the staining viability analysis. After the counting was completed, the hemocytometer was cleaned using isopropanol and DI water and dabbed with a Kimwipe. The hemocytometer was allowed to air dry fully before performing the next measurement. As with THC, the time of the first measurement, before alcohol was introduced, was labeled “$t=-1$”. The start of the staining experiment ($t=0$) was marked when the dye was introduced into the sample.
